# A review of mechanistic and clinical evidence for the use of probiotics and prebiotics in anorexia nervosa

**DOI:** 10.1192/j.eurpsy.2023.1805

**Published:** 2023-07-19

**Authors:** N. Dhopatkar, J. L. Keeler, H. Mutwalli, K. Whelan, H. Himmerich, J. Treasure

**Affiliations:** 1 Eating Disorders, South London and Maudsley NHS Foundation Trust; 2 Institute of Psychiatry, Psychology and Neuroscience; 3 Department of Nutritional Sciences, King’s College London, London, United Kingdom

## Abstract

**Introduction:**

Evidence is growing for the bio-immuno-metabolic model of pathogenesis in anorexia nervosa (AN), an eating disorder with a chronic and relapsing nature. The role of the gut microbiome in this process is also receiving intense research interest. The gut microbiome and the use of probiotics and prebiotics have been extensively studied in gastrointestinal (GI) disorders such as inflammatory bowel disease (IBD) and functional GI disorders (FGIDs). Exploring links between AN and these GI disorders may open new avenues of treatment such as the use of probiotics and prebiotics in AN.

**Objectives:**

This review explores: i) GI presentation in AN and its relationship with the gut microbiome ii) factors influencing the gut microbiome presentation in AN including dietary patterns iii) whether the gut microbiome may be involved in the pathogenesis and maintenance of AN iv) gut microbiome presentation in GI disorders and commonalities with AN v) evidence for the potential use of probiotics and prebiotics as adjunct treatment in AN.

**Methods:**

GI symptomatology and gut microbiome presentation in AN were examined through literature searches. Gut microbiome changes related to common dietary patterns in AN were explored. Microbiome changes that may influence development or maintenance of AN were considered. Microbiome alterations seen in relevant GI disorders were explored and commonalities considered between these and alterations in the microbiome in AN. Literature searches were performed for the use of probiotics and prebiotics in AN and relevant GI disorders.

**Results:**

GI symptoms occur commonly in AN with evidence suggesting some symptoms continuing beyond weight restoration. Significant disruption of the gut microbiome has been associated with AN with some changes related to typical dietary patterns seen during AN development. Additionally, similarities exist between microbiome alterations in AN and those seen in IBD and FGIDs indicating factors apart from the diet, such as a pro-inflammatory milieu, in play. These changes may not only influence GI presentation in AN but may also have a role in maintenance of the disorder. Some evidence suggests that the pre-morbid gut microbiome may influence risk for AN development. Preliminary evidence of the use of probiotics in AN indicates a positive influence on immune modulation although no evidence exists as yet of their influence on AN symptomatology. There has been extensive research into the use of probiotics and prebiotics in IBD and FGIDs with some evidence for reduction in disease parameters and symptomatology with the use of multi-strain probiotics.

**Conclusions:**

Some theoretical, mechanistic and clinical evidence exists for the use of probiotics in ameliorating GI symptoms in AN. However, further research is needed into the context of the gut microbiome changes in AN, the specifics of efficacy and the effects that probiotics and prebiotics may have in AN.

**Disclosure of Interest:**

None Declared

